# Left ventricular dyssynchrony can be observed via cine CMR with use of aortic valve timing

**DOI:** 10.1186/1532-429X-16-S1-P243

**Published:** 2014-01-16

**Authors:** Francisco Contijoch, Kelly Rogers, Walter R Witschey, Robert C Gorman, Yuchi Han

**Affiliations:** 1 University of Pennyslvania, Philadelphia, Pennsylvania, USA

## Background

Cine CMR is the gold standard for evaluation of left ventricular function with high spatial and temporal resolution of the EKG-gated acquisition. Measurements of dyssynchrony have previously been performed using myocardial tagging to measure relative delays in peak shortening of opposing walls. We aim to utilize non-tagged images to quantify dyssynchrony in clinical patients. This is performed by combining aortic valve timing information from a left ventricular outflow tract (LVOT) image series with segmentations of short axis datasets.

## Methods

**Figure 1:**
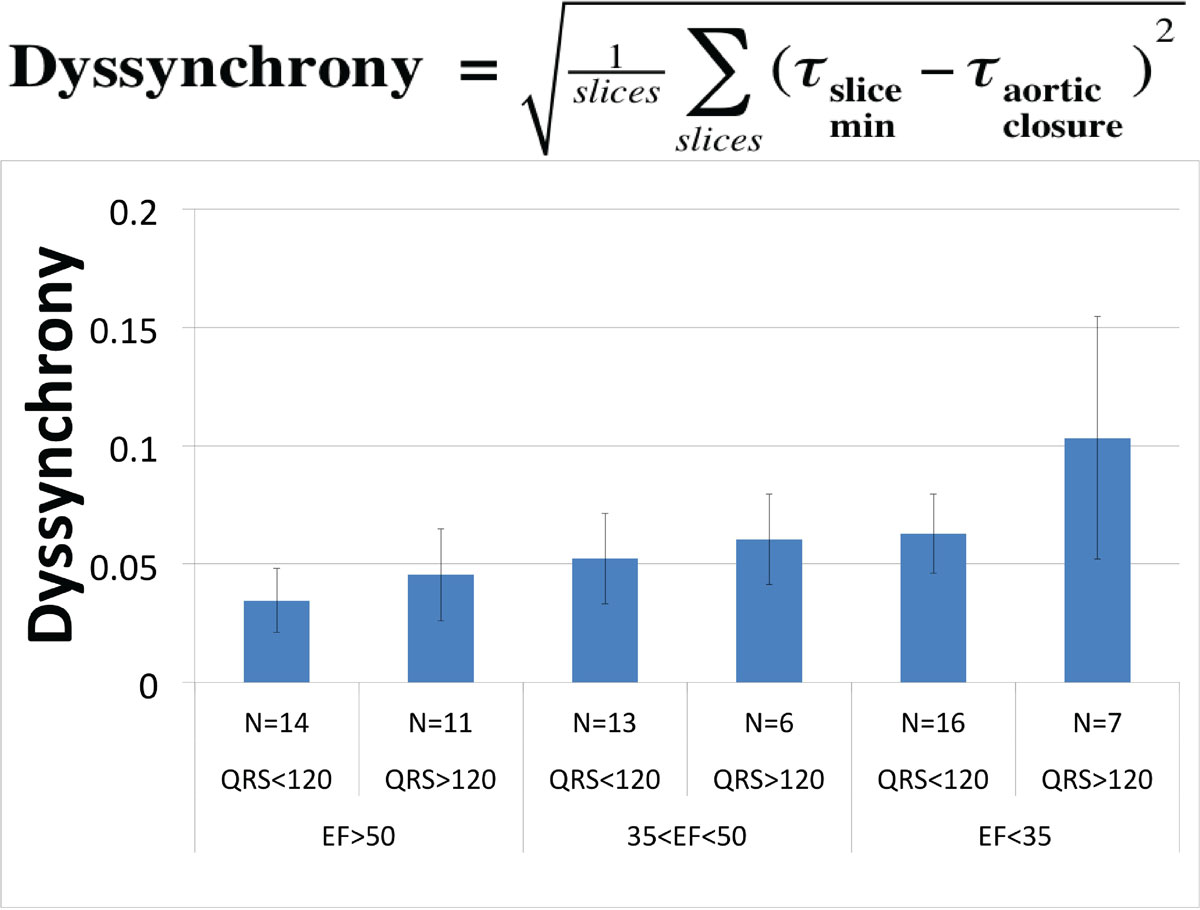
**End-systolic dyssnchrony was quantified in 67 patients with a range of EF and QRS durations**. Observed values were classified based on high, mid and low ED as well as normal prolonged QRS duration. The observed dyssncrony for patients with low EF and prolonged QRS is higher than all other groups. However, more patients are necessary to achieve signifance.

Short-axis stacks and LVOT images were acquired using standard retrospective EKG-gated bSSFP cine imaging in 63 clinical patients with a range of clinical conditions (mean EF = 44.5 ± 19.0%) as well as 29 patients with prolonged QRS duration. Scan parameters were as follows: in-plane resolution: 1.25 - 2.08 mm, slice spacing: 8 mm with 2 mm gap, reconstructed temporal resolution: 18.2 - 58.8 ms. Aortic valve opening and closing as a percentage of cardiac cycle was identified on LVOT images. Volumetric evaluation of short axis images was performed via semi-automated segmentation for all phases and all left ventricular slices (ITK-SNAP, Philadelphia PA). Dyssynchrony was measured by the standard deviation of the timing difference between each slice minimum area to aortic valve closure as expressed by the equation in Figure 1 where τslice min corresponds to the phase at which a slice achieves its minimum area.

## Results

The use of patients with prolonged QRS complexes allows for separation of measured dyssynchrony based on both EF and QRS. Patients were categorized into low EF (≤ 35%), mid EF (35% < EF < 50%) and normal EF (≥ 50%). The results are shown in Figure 1. As has been observed using tagged-MRI, patients with low EF demonstrate higher dyssynchrony than both EF-matched controls as well as patients with long QRS but normal EF.

## Conclusions

This technique allows for a slice-by-slice timing based regional evaluation of dyssynchrony. In addition, subsequent analysis of particular dyssynchronous slices could be performed to provide additional regional information.

## Funding

K99-HL108157, R01-HL103723, T32HL007954, T32-EB009384.

